# Preventing and controlling water pipe smoking: a systematic review of management interventions

**DOI:** 10.1186/s12889-021-10306-w

**Published:** 2021-02-26

**Authors:** Javad Babaie, Ayat Ahmadi, Gholamreza Abdollahi, Leila Doshmangir

**Affiliations:** 1 Department of Health Policy& Management, Tabriz Health Services Management Research Centre, School of Management & Medical Informatics, Tabriz University of Medical Sciences, Tabriz, Iran; 2 Knowledge Utilization Research Center, Tehran University of Medical Sciences, Tehran, Iran; 3 Social Determinants of Health Research Center, Tabriz University of Medical Sciences, Tabriz, Iran

**Keywords:** Management interventions, water pipe, smoking, Tobacco control

## Abstract

**Background:**

Water pipe smoking (WPS) is re-gaining widespread use and popularity among various groups of people, especially adolescents. Despite different adverse health effects of WPS, many of the WPS interventions have failed to control this type of tobacco smoking. This study was conducted to identify experienced management interventions in preventing and controlling WPS worldwide.

**Methods:**

A systematic literature review was conducted. Electronic databases were searched for recordes which were published from beginning 1990 to August 2018. Studies aiming at evaluating, at least, one intervention in preventing and controlling WPS were included in this review, followed by performing the quality assessment and data extraction of eligible studies by two independent investigators. Finally, interventions that were identified from the content analysis process were discussed and classified into relevant categories.

**Results:**

After deleting duplications, 2228 out of 4343 retrieved records remained and 38 studies were selected as the main corpus of the present study. Then, the identified 27 interventions were grouped into four main categories including preventive (5, 18.51%) and control (8, 29.62%) interventions, as well as the enactment and implementation of legislations and policies for controlling WPS at national (7, 25.92%) and international (7, 25.92%) levels.

**Conclusion:**

The current enforced legislations for preventing and controlling WPS are not supported by rigorous evidence. Informed school-based interventions, especially among adolescents can lead to promising results in preventing and controlling WPS and decreasing the effects of this important social and health crisis in the global arena.

**Supplementary Information:**

The online version contains supplementary material available at 10.1186/s12889-021-10306-w.

## Background

Tobacco smoking is one of the main preventable causes of diseases and deaths claiming the lives of 7.2 million annually around the world [1, 2]. Although cigarette smoking is the dominant form of tobacco use in many countries, Water Pipe Smoking (WPS) with other names such as hookah, shisha, narghile, arghile. Goza, oriented pipe, hubble bubble, Mada’s and glaze base, accounts for a significant and growing share of tobacco use globally [3, 4]. In addition, WPS is a culture-based (there are some other types of tobacco smoking behavior) method of tobacco use [5] and its history goes back to 500 years ago in Middle East, North Africa and Asia. However, it has experienced a worldwide re-emergence since 1990 [6] and is regaining popularity among different groups of populations, especially in school and university students [7, 8]. Although WPS is most prevalent in Asia (specifically the Middle East region) and Africa, it has now been changed to a rapidly emerging problem in other continents such as Europe, North, and South America [9, 10]. In recent years, there has been 6–34% increase in tobacco use among 13–15 year olds, most of whom attribute to WPS [10, 11]. In European regions such as Latvia and Czech Republic 22.7 and Estonia 21.9% of people smoke water pipe, while in the Eastern Mediterranean region, the prevalence of WPS is 39.0 and 31.0% of boys and girls, respectively [12]. In average, Lebanon has the highest reported rate (37%) in this regard [12, 13]. In the United States, more than 30% of university students of both genders and 23% of high school students have experienced WPS [14, 15]. Similarly, WPS is also prevalent among highly educated groups. Nearly 20% of health professionals in Jordan and 11% of medical students in London smoked WP [16, 17]. Based on a report, 29.5% of physicians also experience WPS in Pakistan [18].

It has been shown that WPS’ smoking rate can be more addictive compared to that of the cigarette. It also contains more toxic and carcinogenic substances [19, 20] with deleterious effects on the respiratory and cardiovascular systems, as well as oral cavity and teeth [21]. Furthermore, it has a huge negative impact on health costs and the gross domestic product of the countries. For example, the direct and indirect cost of smoking-related diseases is up to $300 billion in the United States annually [22, 23].

Considering the extension of WP businesses, some groups support its expansion [24]. In recent years, the number of WP cafes has increased over many countries. As an example, there are nearly 400 WP cafes in London [25].

Using deceptive advertising, many cafes and restaurants offer WP services along with their orthodox services in order to earn more profit and lure more customers. Moreover, several factors contribute to attracting children and adolescents to WP cafes that leads to an increase in new cases of WPS [26–28]. These factors include the provision of flavored tobacco products or psychotropic WP, the proximity of WP cafe to the public settings such as educational or residential settings, sports clubs, and residential areas, tempting decoration, the provision of study places for students, live music, a variety of games and gambling, and the possibility of watching live movie and sport matches [6, 25, 29, 30].

All this shows that WPS has been turning to a public health crisis. WP business has remained largely unregulated and uncontrolled, which may result in the increasing prevalence of WPS [31]. Moreover, WPS is one of the main factors that can lead to failure in tobacco control [32]. Despite the concerns about WPS outcomes and nearly three decades of using control measures, the prevalence of WPS has increased over the world. Due to the unique nature of WP (multi-components), little is known about the prevention and control of WPS [33]. Thus, special actions and interventions might be required to prevent and control WP tobacco use [33]. Over the recent decade, there has been growing interest among researchers and policymakers regarding addressing the gaps in knowledge about interventions that can be useful in controlling and preventing WPS. Accordingly, this study aimed to identify the management interventions in international and national levels for preventing and controlling water pipe smoking.

## Methods

### Study design

A systematic literature review was conducted. The Preferred Reporting Items for Systematic reviews and Meta-Analysis (PRISMA) guideline [3] was used for performing and reporting the review.

### Inclusion criteria

Primary studies aiming at evaluating, at least, one intervention in preventing and controlling WPS were included.

### Population

WP consumers or people who are likely to be WP consumers in the near future.

### Intervention

Activities, programs, or strategies at the management level aiming at preventing and controlling WP use.

### Outcome

A categorized array of themes presenting a comprehensive picture of management interventions which are targeting WPS prevention and control.

### Search strategy

PubMed, ISI Web of Science, Embase, Scopus, Science Direct, and Ovid were searched for published records from beginning 1990 to August 2018. Further, the first 10 pages of Google Scholar function, World Health Organization (WHO) and World Bank websites were also searched for relevant studies. Additional file 1 provides the terms and search strategy in PubMed.

### Exclusion criteria

Studies were excluded if their focus were on various forms of tobacco use and not just WP use or if they did not distinguish WPS from other forms of tobacco use.

### Quality appraisal

According to the type of the included studies, the critical appraisal checklists of the Joanna Briggs Institute [34] were used for quality appraisal. The Joanna Briggs Institute (JBI) is an international, membership based research and development organization within the Faculty of Health Sciences at the University of Adelaide. JBI Critical appraisal tools have been developed by the JBI and collaborators and approved by the JBI Scientific Committee following extensive peer review. These tools were preliminary for use in systematic reviews. Based on a scoring approach (number of “yes” answers divided by all questions), included studies were categorized to high, moderate, or low quality.

### Data extraction

The data extraction parameters included author, year, country, study design and setting, type of study, participants, the level and type of interventions, study duration, sample size, and main outcomes.

### Data synthesis

Management interventions which influenced controlling and preventing WPS were retrieved and categorized through content analysis method. The interventions were identified and categorized by two researchers (L. D. & J.B) using the following process.

1. Reading the selected records;
2. Identifying and extracting the related interventions after calibration to ensure consistency and accuracy;
3. Grouping the identified interventions into categories and sub-categories based on their conceptual similarity;
4. Solving disagreements between researchers by discussions. Whenever disagreement persisted the third author was approached. In some cases, the identified interventions were placed in more than one category;
5. Confirming categories and subcategories.

## Results

**Fig. 1:**
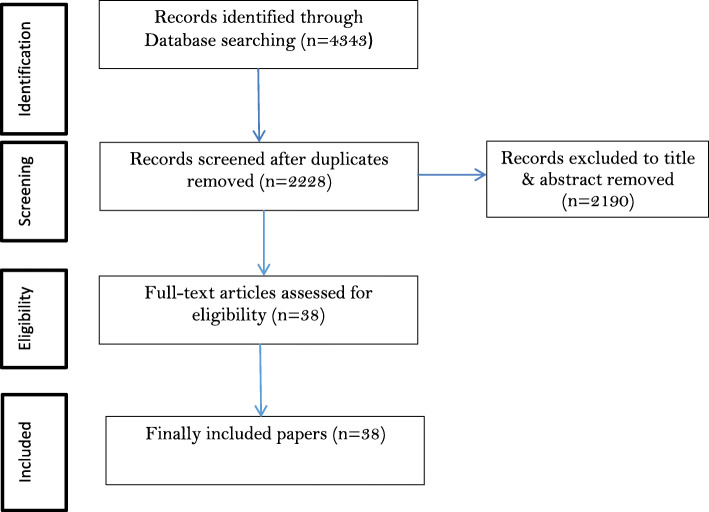
Flow diagram of the search and screening process

The searching process resulted in 4353 studies with 2125(48.81%) of these being repetitions. Out of 2228 screened articles (after removing the duplicates), 38 articles were selected through on the title& abstract screening process. Preferred Reporting Items for Systematic Reviews and Meta-Analyses flow diagram was used to show the number of records in each phase (Fig. 1).

The included studies were of moderate-to-high quality. The characteristics of included studies are provided in Table 1.

**Table 1:** Characteristics of included studies

Author/Year	Country	Design	Setting	Method	Target group	Intervention	Study duration	Sample size	Outcome	Quality
Lock Karen 2010	United Kingdom	Qualitative	Community	Interviews	WP smokers	Smoke-free legislation (SFL)	2007	32	Increase of private smoking	Moderate
Highet G. 2011	United Kingdom	Qualitative	community	Interviews	WP smokers	Implementation of the smoke-free law	April 2007–December 2008	120	Increase of WPS	Moderate
Jawad M. 2013	United Kingdom	Qualitative	universities	Interviews	Regular water pipe smokers	Dispel the misconception about WPS	January–April 2012	32	Decrease of WPS	Moderate
Javad M.2014	United Kingdom	Qualitative	Community	Interviews	local authority staff	Use the experiences of executive authority	May–June 2013	26	Identifying executive problems.	Moderate
Mohd Zin F. 2016	Malaysia	Qualitative	Schools	Semi-structured interviews	Adolescents	Developing new interventions	2015	40	Urgent need to new interventions	Moderate
Grant A.2016	United Kingdom	Exploratory qualitative	Tweets	Documentation	No human subjects	Prevention of web advertising	Jul-05	4439 tweets	WP smoking as an enjoyable activity and a challenge for public health	Moderate
Colditz J. B. 2017	United States	Qualitative (grounded theory)	Web sites	Documentation	No human subjects	Implement of existing tobacco control policies	April–July2013	-	Current interventions are old	Moderate
O’Neill N. 2017	United Kingdom	Qualitative	Email	Modified Delphi Technique	Experts and scientists of behavioral science	Developing of behavior change techniques	Jul-05	24	Effective interventions in quit of WPS	Good
Mostafa A. 2018	Egypt	Qualitative	Community	Interviews	Men and women ≥18	Append of placing pictorial health warnings on WP devices	2015–2016	90	Effective interventions in WPS prevention and stop	Moderate
Anjum Qudsia 2008	Pakistan	Cross-sectional	School	Pre& post-tested	School students 14–19 years old	Health messages	2006	646	Improving knowledge of the students	Moderate
Shishani K. 2011	Jordan	Survey	Hospital	Questionnaire	nurses and physicians	Involving of nurses and physicians’ in WPS control	2010	918	Low incentive and skill to cooperate in WPS control programs.	Moderate
Salti Nisreen 2013	Lebanon	Cross-sectional	Household	Questionnaires	adults	Increasing taxes	2005	13,003	Decrease of WP tobacco demand	Moderate
Ali Quadri M. F.2014	Saudi Arabia	Cross-sectional	Community	Questionnaire	students 15–25 years	Improving the knowledge	2013	1051	Increasing knowledge	Moderate
Kassem N. O. F. 2015	United States	Cross-sectional	University	Questionnaire	Undergraduate Student ≥18	Prohibiting from opening in close to educational places	spring 2007	1332 United States	Effective in WPS	Good
Erdöl C. 2015	Turkey	Survey	Community	Questionnaire	Adults ≥15 years	Increasing of excise taxes and size of pictorial health warnings	2008 and 2012	9030 and 9851	Decrease of WTS	Good
Salloum R. G.2015	United States	Survey	University (Internet-based)	Questionnaire	university students> 18 old & WP smoker	Control fruit-flavored and sweet tobaccos	June–October 2014	367	Decrease of demand for WPS by youth.	Good
Islam F. 2016	United States	Cross-sectional survey	University	Questionnaire	university students smokers > 18	Append of warning labels	June–October 2014	367	Effective to control WPS.	Good
Kingsbury J. H. 2016	United States	Cross-sectional	Community	Questionnaire	Adults≥18	Control of occasional and group smokers	2014	242	Effective to control WPS.	Good
Smith D. M. 2016	United States	Cross-sectional	Telephone-based	Interview	smokers ≥18	Prevention of first use fruit-flavored and sweet tobacco	November 2012–April 2013	1443	More effective in being non user	Moderate
Jaam M.2016	Qatar	Cross-sectional	Community	Interviews	WP smokers	Empowering the families	July–October 2013	181	Decrease of WTS	Moderate
Riggs N. R. 2016	United States	Survey	School base	Questionnaire	School students	Inhibitory Control and Free Lunch	2015	407	Decrease of WPS	Moderate
Jawad M. 2017	Germany	Cross-sectional	WP Fair of International	Observation	Tobacco products	Control of packaging and labelling with guidelines	Jul-05	35	More effective in prevention of WPS	Moderate
Hamadeh R. R. 2017	Bahrain	Cross sectional	Quit clinics	Interview	Male patients smokers	Drugs treatment along with counseling	August–December 2015	194	Effective in quit	Moderate
VanDevanter N.2017	United States	Cross-sectional	Web-based	Questionnaire	nursing students	Training of patients by nurses	February–April 2014	820	Effective in decrease of WPS	Good
Joudrey P. J.2016	US-UAE	Cross-sectional survey	Businesses	Observations and interviews	business owners or managers	control of marketing	January–March 2014.	97	Need to WP-specific legislation.	Moderate
Kowitt S. D.2017	United States	Survey	Community	Checklist	smokers ≥18	Use of FDA Regulation for WP	September 2014 to August 2015	1520	More effective to quit	Good
Deshpande A 2010.	India	Pre & post test	Hospitality venues	(PM2.5) measurements	No human subjects.	Implementation of the smoke free law in hospitality settings	2008–2009	25	Decrease of WPS	Low
Lipk Isaac M. 2011	United States	Randomised controlled	Web-based	questionnaire	University students	Educational interventions of online for colleges’ WP smokers	2009–2010	91	Decrease of WPS	Moderate
Stamm-Balderjahn S. 2012	Germany	Quasi-Experimental	Hospital	Questionnaire	High school students	Educational interventions in clinical settings.	September 2007–July 2008	760	Effective in prevention of smoking	Moderate
Mohlman M. K.2013	Egypt	Quasi-experimental	Community	interviewer	General population	Educational and behavioral interventions	2005–2006	5934	Increase in the attitudes that WP is harmful	Moderate
Asfar T. 2014	Syria	Randomised controlled	clinical		Adults≥18	Brief behavioral interventions clinical settings.	November 2007–October 2008	50	Effective in being none smoking	Moderate
Tomaszek S. 2014	Switzerland	Quasi-Experimental	Hospital	Questionnaire	School students	Brief behavioral interventions by lung specialists.	2009 - February 2013	470	Effective in prevention of school students smoking.	Moderate
Essa-Hadad J.2015	Israel	Quasi-experimental	Web-based	mixed-methods	Students	Web-based education programs.	2007–2010	225	Decrease of WPS	Moderate
Little M. A. 2016	United States	Interventional	Military	questionnaire	Air Force trainees	Brief Tobacco Intervention	October 2014–March 2015	1055	Increase of knowledge	Moderate
Rozema A. D.2018	Dutch	Quasi-experimental	Schools	questionnaire	School students	Outdoor school ground smoking bans	2014–2015	7733	Effective in prevention of WPS	Moderate
Momenabadi V. 2017	Iran	Quasi-experimental	Dormitory	Questionnaire	Students	Educational intervention: BASNEF model	2014	80	Improving of attitudes that WP is harmful	Low
Mahoozi S.2017	Iran	Semi experimental	Medical and hygienic centers	questionnaire	women	Education of women in health center	November 2015–October 2016	60	Improving attitudes that WP is harmful	Moderate
Leavens E. L. S. 2018	United States	RCT	WPS settings	questionnaire	smokers ≥18	Testing exhaled carbon monoxide (CO) before and after and personalized feedback	August–December 2014	109	Effective in quitting WPS	Moderate

The selected studies were published between 1990 and 2018 and focused on 19 different countries including the United States (13.15%) [6, 29, 30, 35, 36], the United Kingdom (7.89%) [25, 37, 38], Germany (5.26%) [12, 39], Iran (5.26%) [40, 41], Egypt [42, 43] (5.26%), Malaysia (2.63%) [44], India (2.63%) [45], Dutch(2.63%) [46], Pakistan (2.63%) [47], Qatar (2.63%) [48], Jordan(2.63%) [16], Lebanon(2.63%) [49], Syria(2.63%) [50], Turkey(2.63%) [51], Bahrain [52] (2.63%), Israel(2.63%) [53], the United Arab Emirates (2.63%) [29], Saudi Arabia [54](2.63%), and Switzerland(2.63%) [55]. Additionally, the type of study design included cross-sectional (31.57%), quasi-experimental (15.78%), and qualitative types (23.68%).

Seventy eight management interventions were identified. After combining interventions with similar concepts into one category, the total number of exclusive interventions condensed to twenty seven.

In the next step, the interventions were assigned to four main subcategories including preventive interventions (18.51%) [12, 35, 40, 44, 46, 48, 54, 56] and control interventions (29.62%) [25, 30, 37, 45, 57, 58], as well as interventions at the international (25.92%) [6, 29, 39, 43, 44, 51, 53, 59, 60] and national (25.92%) [10, 16, 25, 30, 46, 49, 51, 57, 61, 62] levels. The details of the included interventions are presented in Table 2.

**Table 2:** Effective Interventions in Preventing and Controlling Water Pipe Smoking

Main Category	Interventions
Preventive interventions	1-Community-based informing interventions [1–4] 2-College-based education [1, 5, 6] 3-Decreasing social acceptability and occasional smoking [4, 7] 4-Empowering the adolescents and families [8–10] 5-School-based continuous education [11–15]
Control interventions	1-Controlling WP industry marketing [16] 2-Enforcement of new FDA rules [17] 3-Coordinated enforcement of WPS control in adjacent area [18] 4-Involving policymakers to support executive authority in WPS control [19] 5-Licensing and control of all none-WP activities [18] 6-Reducing youth access to WP locations and products [63] 7-Strong implementation of current legislations [20–22] 8-Using successful experiences of authority in WPS control [18]
Enactment and implementation of legislatives and policies on international levels	1-Monitoring activities of WP industry marketing and designing proper control measures [16, 23] 2-Compulsion of industry to append evidence-base health warning labelling in proper places and sizes in WP device, accessories, and other products [24–27] 3-Developing evidence-based control programs tailored to the needs of new generation [9] 4-Encouraging scientists to develop effective interventions of WP control for policymakers [28] 5-Compulsion of industry to decrease the production of various fruit-flavored and sweet tobaccos [29, 30] 6-Preventing social pages and websites from deceptive advertising [31, 32] 7- Developing WP-specific new and clear actions [9, 33]
Enactment and implementation of legislatives and policies on national levels	1-Restricting WP settings [12, 19] 2-Determining proper taxation for WP tobacco packs, devices, and all products [27, 63, 64] 3-Monitoring consumption of medical and nursing students and health care professionals for designing control measures [34] 4-Involving health care professionals to cooperate in the WPS control program [35, 36] 5-Offering evidence-based counseling knowledge about WPS control to health professionals [37] 6-Improving quality of training curricula and informing medical sciences students about WPS control [35] 7-Encouraging executive authorities in developing innovative ways of WPS control [17, 18]

## Discussion

In this study, the management interventions affecting the prevention and control of WPS worldwide were identified through a systematic literature review. In this regard, 27 interventions were experienced in the world for WSP control that was categorized into four main themes and four sub-themes.

### Preventive interventions

Preventive interventions refer to measures that their focus is on abatement of WPS consumption. Some studies suggested that more evidence and investigations are needed to prevent and control WPS [33, 38, 65, 66]. Lopez et al. found that evidence related to WPS control is very rare, and more investigations and studies are required in this respect [33]. Some other studies were related to the current interventions for the prevention and control of WPS that were incompatible with the various needs of the new generations of adolescents. They are poly-users, occasional and social users, and have fast access to new products via the web [5, 44, 67].

To prevent WPS, most studies focused on school-based educational interventions [68]. In many countries, for first time smoking occures in school students and adolescents [69], and students are considered as the current water pipe smokers [12, 41, 46, 48, 54, 55, 70, 71]. For example, the rapidly growing prevalence of experiencing WPS among younger age groups in Lebanon, is going to be considered as an epidemic phenomena [72, 73]. The younger generations have always been lured by fancy advertisements in the media. There have easy access to water pipe bars and are under the illusion and medical myth that the passage of smoke through the water in water-pipes “purifies” the smoke of all harmful elements [74].

NidalEshah et al. (2017) showed that more than 70% of smokers begin WPS in adolescence [75]. In fact, in many countries, young and adolescents’ easy access to café which are providing water pipe facilitates, make them prone to try WPS out [31]. Studies conducted by Aboaziza (2015), Stamm-Balderjahn (2012), and Tugay (2012) revealed that many adolescents become dependent after the first use of WP, which makes the quitting process extremely hard and the educational programs less effective [12, 19, 76]. Thus, access restriction regulations in the time of licensing and controlling their services can be considered as potential intervention.

### Control interventions

Control interventions are activities that try to reduce WPS consumption. The lack of WPS control interventions among students has been reported. Harvey and Phan Thu, P (2016) confirmed that health care professionals have a key role in WPS prevention and control [10, 77]. In a study conducted by Moyer VJP (2013), health care professionals were found to be helping adolescents to change their behavior [78]. On the other hand, Kumar et al.(2015) reported that the prevalence of WPS among health care professionals, especially medical and nursing students [79], can act as a positive vision to WP and cause low motivation to cooperate in WPS control programs [80, 81].

Public education about high-risk behaviors such as WPS was another experienced intervention. Social media, the Internet and mass media are the main factors in promoting or preventing WPS among adolescents.

In recent years, WPS has become a common social behavior and recreation and it is a catering item in many familiar parties. Social acceptance and being an essential part of the family, peer, and public gatherings and café and restaurant culture are highly influential factors contributing to the growth and its popularity. Therefore, exploring the general public’s knowledge and attitude toward WPS is useful in designing and formulating appropriate interventions in controlling WPS [74]. Further, communication and dissemination strategies to facilitate the use of health-related evidence regarding the WPS alongside the role of community health workers, especially in the resource-poor and underprivileged areas of the society and agencies involved in raising public awareness on this issue are essential to be considered [82, 83].

### Enactment and implementation of legislatives and policies in international level

According to the study findings, the WHO Framework Convention on Tobacco Control (FCTC) is a global treaty enacting the actions to control all tobacco products [84]. However, controlling cigarettes and WP-specific actions has received less attention among national policies, and it just has been applied in some studies [85, 86]. It has been shown that using the proposed actions by the FCTC to manage WPS can lead to progress in its prevention and control [51].

Despite the WHO FCTC Article [87] on the taxation of all tobacco products, WP products are still tax-exempt. Although, some studies reported the effectiveness of taxation in reducing tobacco smoking [48, 49, 86], cheap or expensive prices may not be effective in WPS [48, 88]. Several studies suggested that executive authorities have main roles in controlling WPS and should be supported by legislative enforcers and policymakers [25, 30, 89].

According to different studies, the lack of proper interventions in WP industry, including packaging, labeling, advertising, fruit-flavored and sweet tobacco, settings, and diversified services can lead to a failure in WPS control programs [6, 29, 30, 43, 44, 59, 60]. Other studies represented that there is a strong relationship between fruit-flavored and sweet WP tobacco products and the expansion of WP use and act as the main barrier for WPS control [90, 91]. Therefore, measures to ban these additives proposed to be considered [6, 92, 93]. Furthermore, previous evidence shows that proper warning labels accompanied by a clear and intelligible packaging can be more effective in controlling WPS [25, 43, 51, 94, 95].

### Enactment and implementation of legislatives and policies in national level

Although there are extensive WPS restrictive rules in countries [65], the lack of coordination between the involved organizations and the lack of executive support have led to their inefficiency [96]. Community involvement and advocacy were found among the strongest WPS prevention measures [97, 98]. Moreover, community representatives, local and identical groups, and local community centers advocacy had shown some extend of efficacy as management interventions [99].

Some researchers believe that smoking related harms could not be completely prevented. Therefor harm reduction strategies were proposed in studies [100]. Although, those strategies might be interesting for cigarette, they do not necessarily applicable for hookah [101]. Recently, three harm reduction components (quick-light charcoal, electric heating and bubble diffuser quick-light charcoal and bubble diffuser) have been examined [102], however such strategies are not yet agreed upon and needs some more evidences [100].

### Strengths and limitations of the study

Although this study was not aimed to evaluate interventions and provide some information about their efficacy, summarizing the intervention effects across themes would be valuable. However, we could not find well-defined interventional studies using a common evaluation means. Additionally, most found interventions were complex interventions with a variety of components making the synthesis of intervention effects more challenging.

## Conclusion

In general, our findings indicated WPS related social and health crisis have not come into attention in high levels of decision making. The current enforced legislations are old, unclear, and incompatible with the needs of the adolescents and are not backed by rigorous evidence. In addition, the WP industry is rapidly expanding without monitoring and controlling measures. Informing and empowering adolescents for those who have not yet experienced smoking is a sensible intervention in this regard. Besides, empowering and involving health students and professionals in WPS control programs can lead to promising results in preventing and controlling WPS. It seems that there is a paucity of evidence regarding strategies on controlling and preventing WTS, thus further research in the society is warranted in this respect.

## Supplementary Information

**Additional file 1.**

## Data Availability

All of the included papers are available in PubMed, ISI Web of Science, Embase, Scopus, Science Direct, and Ovid databases.

## Abbreviations

WPS: Water Pipe Smoking
WP: Water Pipe
GDP: Gross Domestic Product
WHO: World Health Organization
FCTC: Framework Convention on Tobacco Control
