# Supplementary material for: Preventing and controlling water pipe smoking: a systematic review of management interventions
Source: BMC Public Health. 2021 Feb 26;21:344. doi: 10.1186/s12889-021-10306-w (PMC7908788; doi:10.1186/s12889-021-10306-w)
Supplement: Supplementary file 1 — Additional file 1. [file 12889_2021_10306_MOESM1_ESM.docx]

**Appendix 1: The terms and search strategy for PubMed database**

**A:** Search (((((((((((((((((((((((((((((((((("Water pipe"[Title/Abstract]) OR "Water-pipe"[Title/Abstract]) OR "Waterpipe"[Title/Abstract]) OR "Hubble bubble"[Title/Abstract]) OR "Hubble-bubble"[Title/Abstract]) OR "Hubblebubble"[Title/Abstract]) OR Arghile[Title/Abstract]) OR Argileh[Title/Abstract]) OR Arguileh[Title/Abstract]) OR Argeeleh[Title/Abstract]) OR Argela[Title/Abstract]) OR Argila[Title/Abstract]) OR Narghile[Title/Abstract]) OR Narghileh[Title/Abstract]) OR Narguileh[Title/Abstract]) OR Narguile[Title/Abstract]) OR Nargila[Title/Abstract]) OR Nargile[Title/Abstract]) OR Nargileh[Title/Abstract]) OR Qalyan[Title/Abstract]) OR Hookah[Title/Abstract]) OR Huqqa[Title/Abstract]) OR Hukkah[Title/Abstract]) OR Hukka[Title/Abstract]) OR Goza[Title/Abstract]) OR Borry[Title/Abstract]) OR Sheesha[Title/Abstract]) OR Shisha[Title/Abstract]) OR Shesha[Title/Abstract]) OR Shishe[Title/Abstract]) OR Chicha[Title/Abstract]) OR Mada’a[Title/Abstract]))

AND

**B**: Search ((((control*[Title/Abstract]) OR prevent*[Title/Abstract]) OR cessation*[Title/Abstract]) OR quit*[Title/Abstract]) OR reduct*[Title/Abstract]

AND

**C**: Search ((((((((((((((((((((((((((((((((((manage*Title/Abstract]) OR strategy [Title/Abstract]) OR strategies [Title/Abstract]) OR policy [Title/Abstract]) OR policies [Title/Abstract]) OR intervention* [Title/Abstract]) OR practice [Title/Abstract]) OR legislation* [Title/Abstract]) OR decision* [Title/Abstract]) OR option* [Title/Abstract]) OR plan* [Title/Abstract]) OR law* [Title/Abstract]) OR rule* [Title/Abstract]) OR opportunity* [Title/Abstract]) OR action*[Title/Abstract]) OR activities*[Title/Abstract]
